# The prognostic role of circulating CD8^+^ T cell proliferation in patients with untreated extensive stage small cell lung cancer

**DOI:** 10.1186/s12967-019-02160-7

**Published:** 2019-12-03

**Authors:** Ning An, Haoyi Wang, Wenxiao Jia, Wang Jing, Chao Liu, Hui Zhu, Jinming Yu

**Affiliations:** 1 Department of Radiation Oncology, Shandong Cancer Hospital and Institute, Shandong University, Jinan, China; 2 Department of Hematology, Qilu Hospital, Shandong University, Jinan, China; 3 Department of Radiation Oncology, Shandong Cancer Hospital and Institute, Shandong First Medical University and Shandong Academy of Medical Sciences, Jinan, China

**Keywords:** Small cell lung cancer, Peripheral lymphocytes, Immunosuppression, CD8^+^ T cells

## Abstract

**Background:**

Immunosuppression caused by tumorigenesis may promote tumor progress and invasion. Here, we investigated whether the characteristics of circulating T lymphocyte subtypes in patients with extensive small cell lung cancer (ED-SCLC) can be used as an alternative marker of tumor progression.

**Methods:**

This study included 36 newly diagnosed ED-SCLC patients before treatment and the patients were followed up. 22 age and sex-matched healthy volunteers were selected as control. The percentages and proliferation potential of T lymphocyte subpopulations from peripheral blood were measured.

**Results:**

CD4^+^ CD25^+^ Foxp3^+^ regulatory T cells (Tregs) were elevated in ED-SCLC patients compared with healthy controls (*p *= 0.0083). In contrast, the percentages of CD3^+^ and CD3^+^CD4^+^ T cells were significantly lower in SCLC patients (*p *< 0.001; *p *= 0.0014). The proliferation (%divided) of CD8^+^ T cells of SCLC patients was suppressed compared with healthy controls (*p *= 0.0058), but not of CD4^+^ T cells (*p *= 0.1611). Multivariate analyses showed that the %divided of CD8^+^ T cells is an independent predictor for PFS (HR: 4.342, 95% CI 1.324–14.245; *p *= 0.015). The percentages of peripheral Tregs and the degree of chemotherapy or radiotherapy induced lymphopenia negatively correlated with the proliferation of CD8^+^ T cells (*p *= 0.0225, r = − 0.379; *p *= 0.0003, r = − 0.464).

**Conclusion:**

The present study indicates that SCLC patients have impaired immunity in peripheral blood, and the proliferation potential of circulating CD8^+^ T cells is a significant predicator for PFS.

## Background

Small cell lung cancer (SCLC) accounts for 10% to 15% of all lung cancer, with rapid growth and extensive metastasis [1, 2]. The high invasiveness of SCLC is thought to be associated with its high mutation burden, such as the inactivation of the tumor suppressor gene p53 in almost the whole tumor [3]. About 70% of patients suffer from the extensive disease at the time of diagnosis [4]. In recent decades, there was no significant change in the systematic treatment of patients with SCLC. Chemotherapy was still the standard treatment with no new drugs were introduced. SCLC is highly sensitive to first-line chemotherapy. However, about 80% of patients with limited-disease SCLC and almost all patients with ED-SCLC eventually recur and develop into progressive disease [5, 6].

While conventional treatments of SCLC generally provide limited clinical benefits, there is evidence that immunotherapy is becoming more and more promising for the disease [7]. A few patients with SCLC have immune responses to tumor-associated antigen, and the prognosis is better [8]. Meanwhile, most SCLC patients have low immune functions with local and systemic immune deficiencies, which are associated with worse morbidity and mortality [9, 10]. Immune evasion has been identified as a fundamental feature of cancer [11]. It has been demonstrated the interaction between tumor cells and the host immune system is important in regulating tumor growth [12]. Tumor cells evade immunosurveillance by several mechanisms [13].

T lymphocyte can respond to many types of human cancer, especially those with a high mutation burden [14]. T cell exhaustion (T_ex_) was first identified in chronic virus infection, which was defined as antigen-specific T cell deficiency or persistence of adverse effects and proliferation [15]. Subsequently, it was quickly realized that CD8^+^ T cell exhaustion also existed in cancer [16, 17]. Compared with effector and memory CD8^+^ T cells, T_ex_ cells had weaker effector function and experienced a different pattern of differentiation [18]. T_ex_ cells are also actively suppressed by inhibitory receptors, such as programmed death-1 (PD-1) [17]. The PD-1 blocker can partially activate T_ex_ cells in preclinical models [19], which has led to clinical benefits in a lot of human cancers [13].

However, the frequent detection of lymphocytes for tumor antigens is limited to tumor-infiltrating lymphocytes recently [20]. Besides, it is indistinct whether circulating lymphocytes can be used to monitor responses to treatment, identify associated reactive cell types, and allow for an in-depth understanding of the potential immunological mechanisms of ongoing clinical responses. Furthermore, immune landscape profiles are largely unknown in patients with SCLC, and these changes may interfere with prognosis and treatment responses.

In this study, we investigated the presence of immune landscape markers (lymphocyte subtypes and proliferation) in newly diagnosed SCLC patients and followed up the prognosis of these patients. Considering the major role of CD8^+^ T lymphocytes in antitumor immunity, we focused on CD8^+^ T cells to identify the potential biomarkers of prognosis.

## Methods

### Participants

A cohort of patients with initially diagnosed ED-SCLC was enrolled into the study at the Shandong Cancer Hospital from May 2018 to May 2019. Eligible patients were diagnosed as SCLC by pathological biopsies and imaging staging (CT or PET-CT, MRI). The state of (ECOG) was 0–2 in the eastern cooperative tumor group. Exclusion criteria included the presence of a hematological disease or immune deficiency and participation in vigorous exercise within 24 h before sampling. All patients were sampled before tumor treatment. The control group passed a comprehensive medical evaluation before being included in the study. This study was approved by the Medical Ethics Committee of Shandong Cancer Hospital, and all participants provided written informed consent.

### Lymphocytes isolation and culture

Ficoll–Hypaque centrifugation (Amersham Biosciences, Piscataway, NJ, USA) was used to isolated peripheral blood mononuclear cells (PBMCs) of SCLC patients and healthy controls. The isolated cells were resuscitated in RPMI-1640 medium (Life Technologies, Paisley, UK), supplemented with 10% heat-inactivated fetal bovine serum (Gibco, Grand Island, NY, USA), 1% penicillin and streptomycin (Solarbio, Beijing, China). Co-cultured with recombinant human IL-2 (10 ng/mL, R&D Systems, Minneapolis, MN, USA), anti-human CD3 antibody (1 ng/mL; eBioscience, San Diego, CA, USA) and anti-human CD28 antibody (1 ng/mL; eBioscience).

### Evaluation of proliferation capacity of lymphocytes via %divided calculations

The isolated cells were labeled with 5,6-carboxy fluorescein diacetate succinimide ester (CFSE, 5 µmol/L; Sigma‐Aldrich). In short, CFSE was added to the cell suspension and incubated at 37 °C for 15 min, then 3 mL fetal bovine serum was added and placed at 4 °C for 5 min to stop the reaction. The CFSE labeled cells were incubated with rhIL-2, CD3 and CD28 in RPMI-1640 medium for 5 days, and then stained with PE-cy7-anti CD4, APC-anti CD8, APC-cy7-anti CD3 antibody (eBioscience) according to the manufacturer’s instructions.

The %divided was calculated by analyzing the CFSE profile of the populations of interest with the Proliferation Platform of Flow Jo FACS Analysis Software (Tree Star, Inc., Ashland, OR). The %divided is the percentage of the cells of the original sample that divided, which reflect the proliferation potential of the original cell population. It is the same as the Precursor frequency. The calculation of %divided (precursor frequency) was described previously [21, 22]. The CFSE fluorescence intensity of cells divided once shows a half value of CFSE-fluorescence intensity of the undivided cells. Divisions of reactive cells, which were identified and determined by their CFSE intensities, were labeled from 0 to n as division round. A single cell dividing n times will generate 2n daughter cells. With use of this mathematical relationship, the number of division precursors was extrapolated from the number of daughter cells of each division and from proliferation events. The %divided was calculated by dividing the sum of precursor T cells that had divided once or more by the sum of all precursor T cells, including the precursor T cells that had not divided.

### Flow cytometry analysis of the percentages of Tregs and CD4^+^/CD8^+^ T subsets

**Fig. 1:**
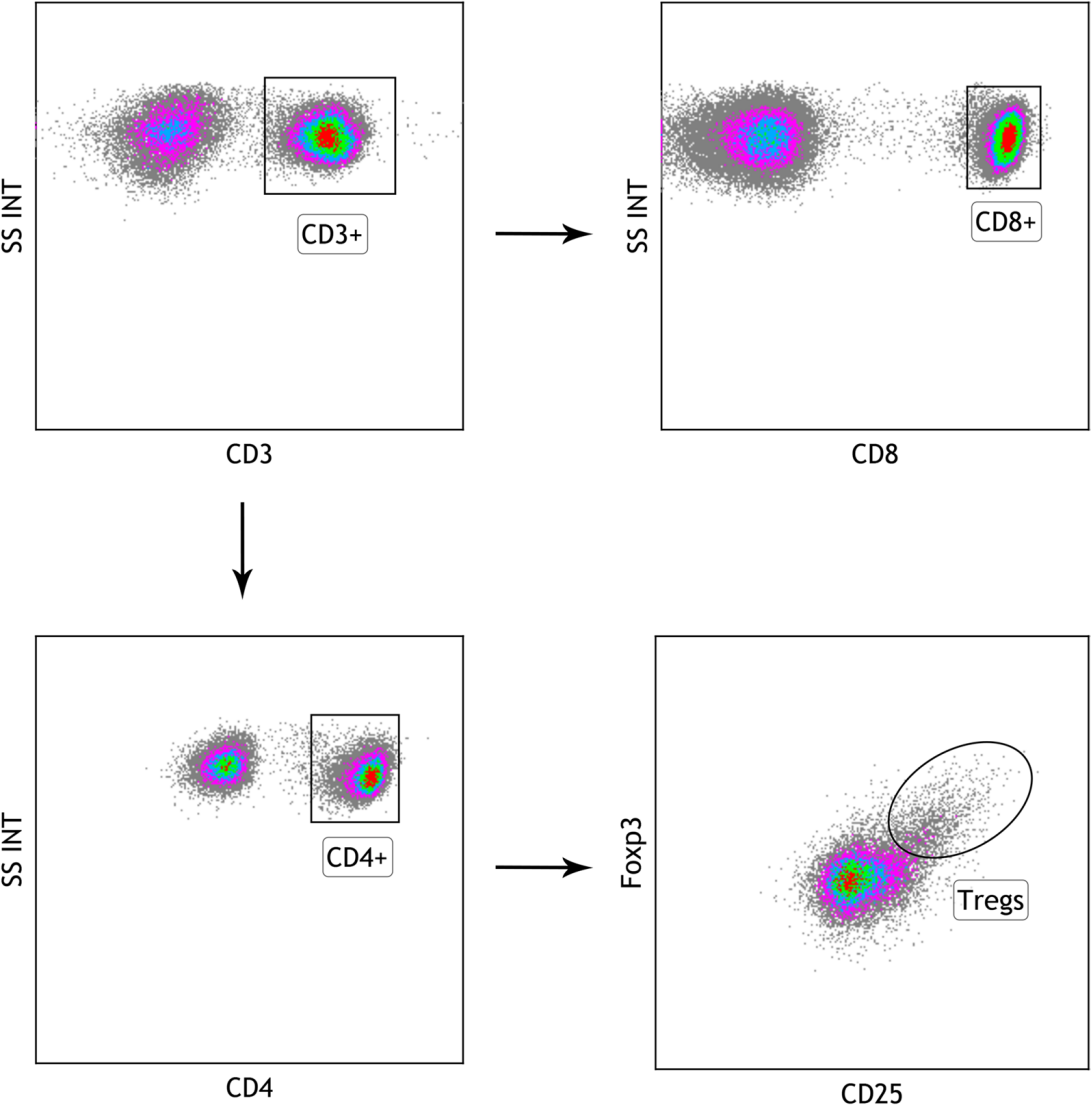
Typical flow cytometry plots and gating for CD3^+^, CD3^+^CD4^+^, CD3^+^CD8^+^ and CD4^+^CD25^+^Foxp3^+^ Treg cells

PBMCs were isolated and stained with a panel of antibodies on surface. Briefly, incubate about 1 × 10^6^ PBMCs in 100 μL of 1% PBS-BSA with appropriate combination of antibodies as per manufacturer’s instructions at room temperature in the dark. Then, wash the cells with PBS and resuspend the cells in 500 μL of PBS for flow cytometric analysis. For all samples, 100,000 events were acquired for analysis in an eight-colour flow cytometer (Beckman Coulter, Analis, Belgium). FACS Calibur cytometer equipped with Kaluza Flow Cytometry Analysis Software (Beckman Coulter, Analis, Belgium) was used to carry out data analysis. Tregs were stained using FITC-anti CD4, PE-anti CD25, and APC-anti Foxp3 antibodies, and T subsets were detected with FITC-anti CD4, APC-anti CD8, and APC-cy7-anti CD3 antibodies (eBioscience). Typical flow cytometry plots and gating for CD3^+^, CD3^+^CD4^+^, CD3^+^CD8^+^ and CD4^+^CD25^+^Foxp3^+^ Treg cells are shown in Fig. 1.

All antibodies were matched with isotype controls (Santa Cruz Biotech, Heidelberg, Germany). In order to eliminate autofluorescence, fluorochrome interferences and dead cells, quality control panels were used including compensation controls based on single fluorochrome staining, fluorescence-minus-one controls and dead cell exclusion control. Fluorescence-minus-one controls include other stains and exclude the stain in a particular channel to define the boundary between positive and negative cells. Machine quality controls were performed daily.

### Statistical analysis

Continuous variables were compared using Student’s *t* test or the Mann–Whitney U test. Univariate analysis and multivariate analysis used to detect independent predictors of PFS were based on the Cox proportional hazards regression model. The factors with p values of < 0.10 on univariate analysis were entered into the multivariate analysis. The PFS curves were calculated according to the Kaplan–Meier method and compared using the log-rank test. Linear correlations between Tregs, CD3^+^, CD4^+^, CD8^+^, NLR, PLR and the proliferation of CD8^+^ T cells were based on the Spearman correlation coefficient, and the correlations between the degree of lymphopenia and the proliferation of CD8^+^ T cells were based on the Kendall correlation coefficient. Statistical significance was defined as p < 0.05 with 2 sides. SPSS 20.0 (Armonk, NY, USA) and Graphpad Prism 7 (GraphPad Software, San Diego, CA) was used to conduct statistical analyses.

## Results

### Patients

36 patients with SCLC (63.5 years [20–85]; 28 males, 8 females) were included in the study before treatment. 22 age and sex-matched healthy persons were also included as controls. The media follow up time was 10.7 months and the median PFS of the SCLC patients was 4.0 months. The patient’s characteristics and treatment details are listed in Table 1.

**Table 1:** Patients characters

	N (36)	%
Age (median, range), years	63.5 (20–85)	
Sex		
Male	28	77.8
Female	8	22.2
Metastatic site		
Brain	5	21.74
Lung	5	21.74
Bone	7	30.43
Liver	5	21.74
Distant lymph node	5	21.74
Kidney	2	8.70
Adrenal gland	3	13.04
First-line treatment		
Etoposide and platinum	27	75
Etoposide and platinum + radiotherapy	9	25

### Percentages of circulating lymphocyte subsets in SCLC patients and healthy controls

**Fig. 2:**
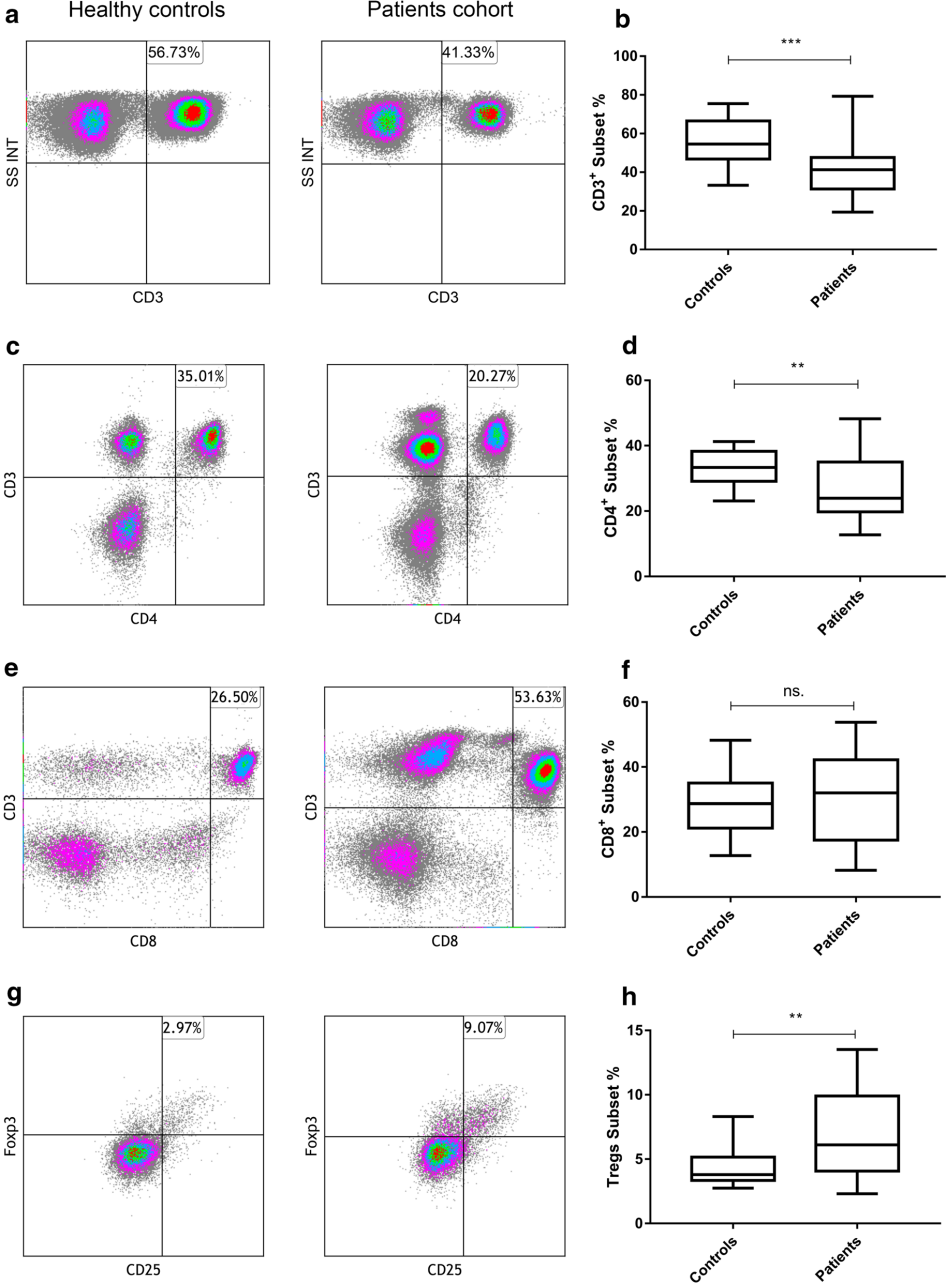
Percentages of lymphocyte subsets in peripheral blood. **a**, **b** The percentages of peripheral CD3^+^ subsets are lower in SCLC patients than healthy controls, 41.9 ± 14.5 vs. 55.6 ± 11.8, *p *< 0.001; **c**, **d** CD3^+^CD4^+^ subsets are lower in SCLC patients, 27.0 ± 9.43 vs. 33.5 ± 5.08, *p *= 0.0014; **e**, **f** there is no significant statistic difference of CD3^+^CD8^+^ subsets between the SCLC patients and controls, 30.9 ± 12.8 vs. 28.8 ± 9.97, *p *= 0.4914; **g**, **h** the percentage of CD4^+^CD25^+^Foxp3^+^ subsets is elevated of SCLC patients compared to that of healthy controls, 6.11 [2.13–13.5] vs. 3.8 [2.74–8.30], *p *= 0.0083. Differences between two groups were determined by Student’s t test or Mann–Whitney test (Additional file 1: Table S1)

It has been reported that the ratio of different subsets of circulating lymphocytes was alerted in cancer patients [23, 24]. To avoid the effects of chemotherapy or radiotherapy on blood lymphocytes, we selected patients who had not received any treatment at the first diagnosis. The proportion of CD3^+^, CD3^+^CD4^+^, CD3^+^CD8^+^, and CD4^+^CD25^+^Foxp3^+^ Treg cells in the peripheral blood of SCLC patients were compared to those in healthy controls. Significant differences were observed in the percentages of CD3^+^, CD3^+^CD4^+^ and CD4^+^CD25^+^Foxp3^+^ Treg cells. The percentage of CD4^+^CD25^+^Foxp3^+^ Tregs subset was increased in SCLC patients compared to that of healthy controls (6.11 [2.13–13.5] vs. 3.8 [2.74–8.30], *p *= 0.0083, Fig. 2g, h). By comparison, the proportion of CD3^+^ and CD3^+^CD4^+^cells were significantly lower in SCLC patients than controls (41.9 ± 14.5 vs. 55.6 ± 11.8, *p *< 0.001, Fig. 2a, b; 27.0 ± 9.43 vs. 33.5 ± 5.08, *p *= 0.0014, Fig. 2c, d). However, CD3^+^CD8^+^ T lymphocyte subsets have no difference between the patients and controls (30.9 ± 12.8 vs. 28.8 ± 9.97, *p *= 0.4914, Fig. 2e, f).

### The proliferation potential of circulating lymphocyte subsets in SCLC patients and healthy volunteers

**Fig. 3:**
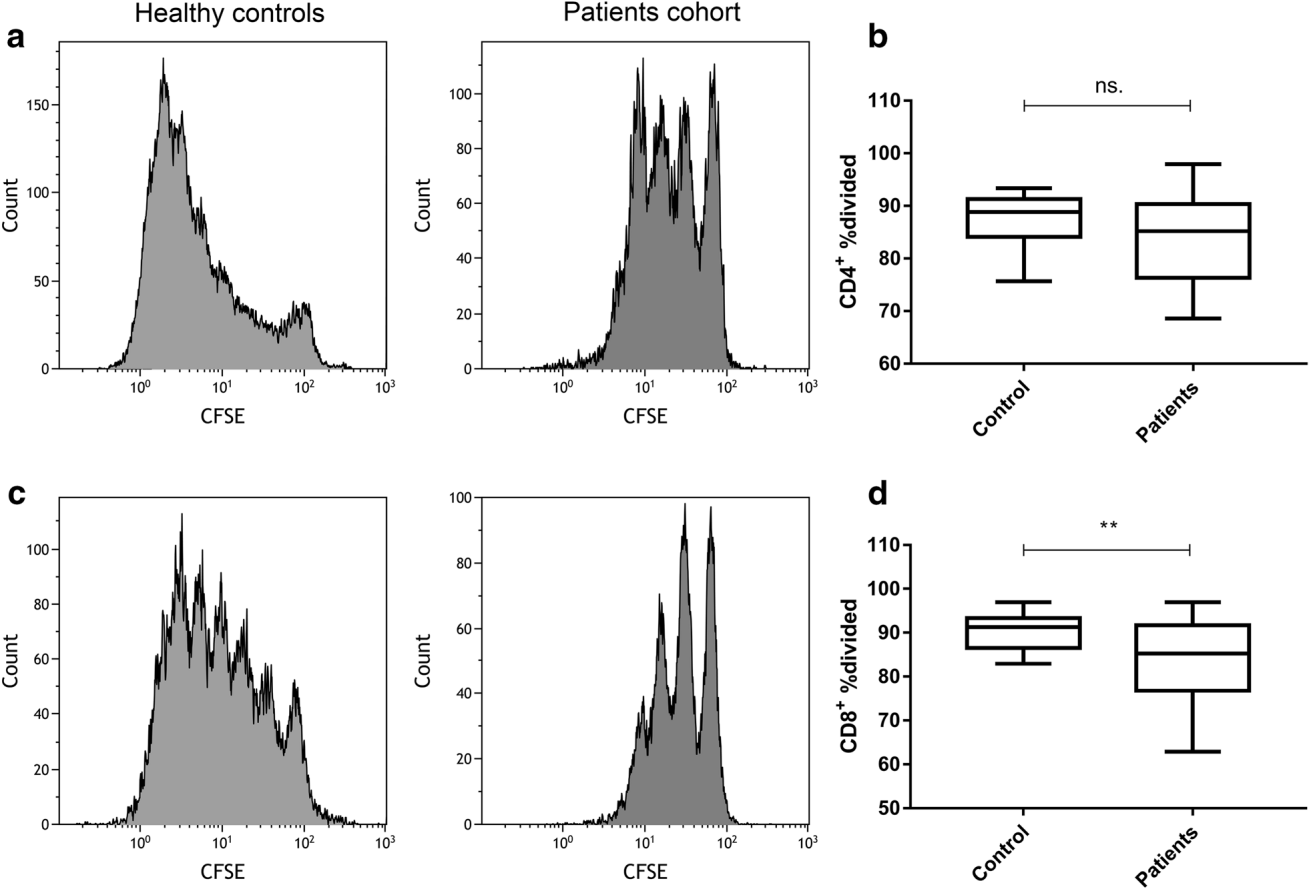
The proliferation potential of different subsets of peripheral T cells. **a**, **b** The %divided of CD8^+^ T cells of SCLC patients was lower compared with healthy controls, 85.3 [62.9–96.3] vs. 91.3 [82.9–96.9], *p *= 0.0058; **c**, **d** the mean %divided of CD4^+^ T cells of SCLC patients was lower than that of healthy controls, but no statistic difference was observed, 85.2 [68.6–97.9] vs. 88.8 [75.6–93.4], *p *= 0.1611. Differences between two groups were determined by Mann–Whitney test (Additional file 1: Table S1)

CFSE was used to stain the surface of living cell membranes. As the cells divided, the content of CFSE on the surface of the cell membrane also decreased, and the mean fluorescence intensity also decreased. A group of cells with the strongest fluorescence intensity represents primary cells. The %divided is the percentage of the cells of the original sample that divided, which reflect the proliferation ability of the original cell population. It was calculated by analyzing the CFSE profile of the populations of interest with the Proliferation Platform of Flow Jo FACS Analysis Software. The proliferation of CD8^+^ T cells of SCLC patients was significantly suppressed compared with healthy controls (85.3 [62.9–96.3] vs. 91.3 [82.9–96.9], *p *= 0.0058, Fig. 3a, b). The mean %divided of CD4^+^ T cells of SCLC patients was lower than that of healthy controls, but we did not observe statistic difference (85.2 [68.6–97.9] vs. 88.8 [75.6–93.4], *p *= 0.1611, Fig. 3c, d).

### Correlation of independent variables with PFS

**Fig. 4:**
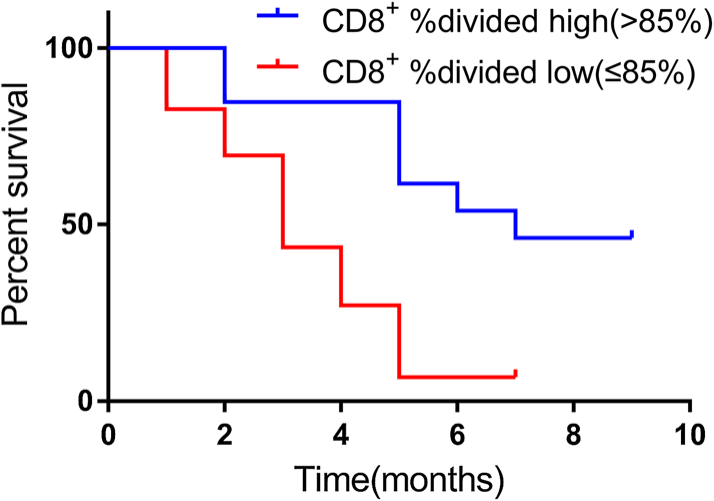
Kaplan–Meier curve of PFS for patients with different %divided of CD8^+^ T cells. The median PFS in patients with high %divided of CD8^+^ T cells subset percentage (≥ 85.0%) was significantly benefit compared with that in patients with low %divided of CD8^+^ T cells subset percentage (< 85.0%), 3.0 vs. 7.0 months HR: 0.3247, 95% CI 0.1503 to 0.7014, *p *= 0.0016, log-rank test

Correlative studies were performed to investigate the correlation between circulating biomarkers and response to standard therapy. The cutoff values of these biomarkers were determined based on the near medians. The Cox regression analysis was used to test the prognostic value of independent variables in univariate analyses (Table 2). Four factors (*p *< 0.1) were included in multivariate analyses. The proportion of CD3^+^ T lymphocytes and the %divided of CD4^+^ and CD8^+^ T cells positively correlated with PFS, and the neutrophil to lymphocyte ratio(NLR) negatively associated with PFS (Table 2). However, only the %divided of CD8^+^ T cells was an independent predictor associated with PFS in the multivariate analyses (Table 3). The PFS curve for patients with different %divided of CD8^+^ T cells was calculated by Kaplan–Meier methods (Fig. 4). The median PFS of patients with high CD8^+^ %divided (> 85%) was significantly longer than that in patients with low CD8^+^ %divided (≤ 85%) (3.0 vs. 7.0 months HR: 0.3247, 95% CI 0.1503 to 0.7014, *p *= 0.0016, Fig. 4).

**Table 2:** Univariate analysis for PFS

Variable	Univariable analysis		
	HR	95% CI	*p*
Age			
> 65 vs. < 65	1.161	0.512–2.638	0.720
Sex			
Male vs. female	1.589	0.588–4.296	0.361
Smoking status			
Smoker vs. never-smoker	0.970	0.410–2.293	0.945
Intracranial metastasis			
Yes vs. no	1.398	0.469–4.173	0.548
Treatment			
EP vs. EP + radiation	0.710	0.263–1.914	0.499
Lymphopenia			
Yes vs. no	1.488	0.656–3.376	0.342
NLR			
< 5.691 vs. > 5.691	0.406	0.149–1.107	0.078
PLR			
< 186.26 vs. > 186.26	0.861	0.380–1.953	0.721
CD3^+^			
< 38.90 vs. > 38.90	2.359	0.968–5.946	0.059
CD3^+^ CD4^+^			
< 23.90 vs. > 23.90	0.935	0.409–2.137	0.874
CD3^+^ CD8^+^			
< 32.00 vs. > 32.00	1.098	0.475–2.539	0.827
CD4^+^ CD25^+^Foxp3^+^			
< 6.111 vs. > 6.111	1.431	0.625–3.274	0.396
CD4^+^ %divided			
< 84.40 vs. > 84.40	2.306	0.962–5.527	0.061
CD8^+^ %divided			
< 85.00 vs. > 85.00	5.252	1.724–16.000	0.004

**Table 3:** Multivariable analysis for PFS

Variable	Multivariable analysis		
	HR	95% CI	*p*
NLR			
< 5.691 vs. > 5.691	0.449	0.157–1.287	0.136
CD3^+^			
< 38.90 vs. > 38.90	1.638	0.634–4.230	0.308
CD4^+^ %divided			
< 84.40 vs. > 84.40	1.577	0.629–3.955	0.331
CD8^+^ %divided			
< 85.00 vs. > 85.00	4.342	1.324–14.245	0.015

### Correlations between other plasma biomarkers and the proliferation of CD8^+^ T cells

**Fig. 5:**
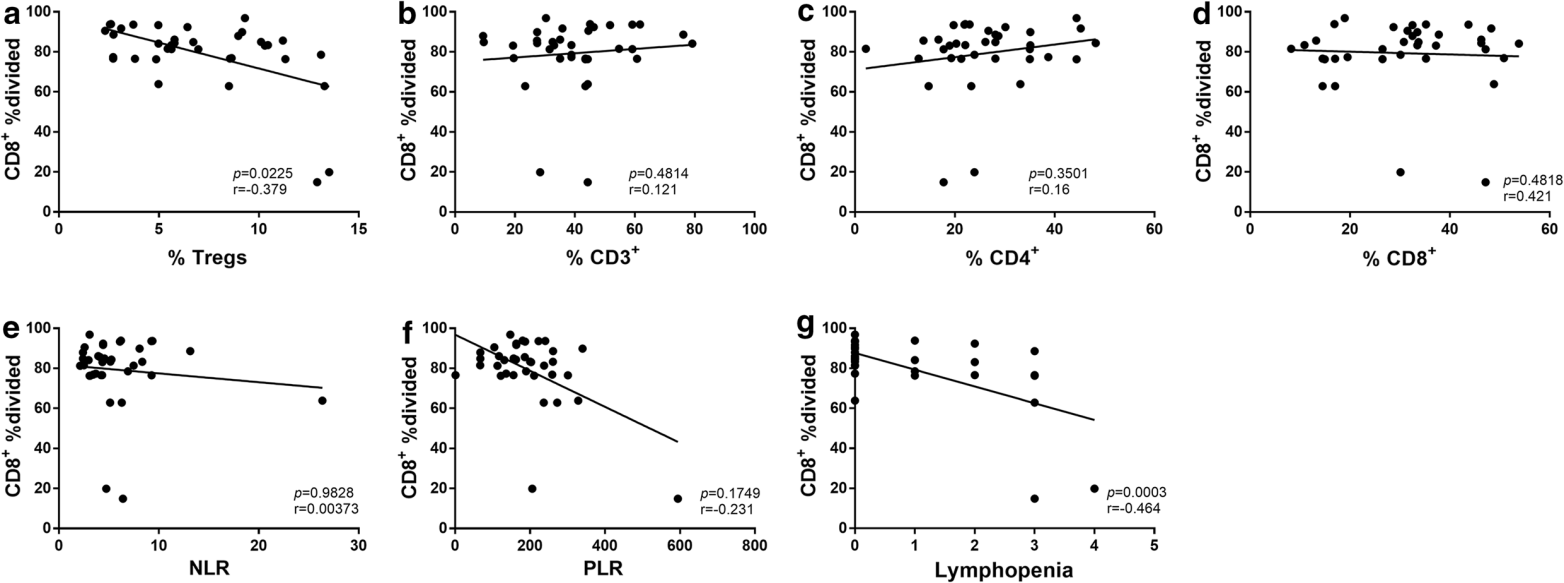
Correlations between different plasma biomarkers and the proliferation of CD8^+^ T cells. **a** peripheral Tregs subsets negatively correlated with the %divided of CD8^+^ T cells, *p *= 0.0225, r = − 0.379; **g** the degree of chemotherapy or radiotherapy induced lymphopenia were negatively correlated with the proliferation of CD8^+^ T cells, *p *= 0.0003, r = − 0.464; **b**–**f** no significant correlations were observed between other circulation T cells subsets or NLR and the proliferation of CD8^+^ T cells

The proliferation of CD8^+^ T cells may correlate with patients’ clinical characters. The results of our study indicated that peripheral Tregs subset negatively correlated with the proliferation of CD8^+^ T cells (*p *= 0.0225, r = − 0.379, Fig. 5), but no significant correlations were observed between other circulation T cells subsets and the proliferation of CD8^+^ T cells (Fig. 5). The degree of chemotherapy or radiotherapy induced lymphopenia (CTCAE 5.0) was negatively correlated with the proliferation of CD8^+^ T cells (*p *= 0.0003, r = − 0.464, Fig. 5). Although NLR was reported to be associated with the prognosis of cancer patients, we didn’t observe the correlation between it and the proliferation of CD8^+^ T cells.

## Discussion

To the best of our knowledge, the present study is the first to investigate the relationship between the proliferation of peripheral blood T cells and the prognosis of patients with SCLC. We found that peripheral CD8^+^ T cell proliferation was inhibited in untreated SCLC patients. This study revealed the independent predictive value of the circulating CD8^+^ T cell proliferation potential in patients with SCLC.

Immune dysfunction is common in most malignant tumors [25], indicating that immunosuppression may associate with the progress of cancer and the treatment of drug resistance. Increasing evidence has suggested the prognostic value of tumor-infiltrating immune cells [26, 27]. Tumor biomarkers from different sources have been widely used to assess clinical responses to treatment and disease progress or recurrence. However, the biomarkers currently in use may only reveal late events of tumor progress with little help in reflecting potential early changes. Whether the features of circulating lymphocyte subsets have prognostic or predictive value is not clear [28–30]. There are no reliable parameters that predicting the benefits of the treatment because the assessment of tumor response only reflects the efficiency of anti-tumor therapy after a few cycles. Therefore, to determine the predictive value of circulating lymphocytes on the disease status and PFS of SCLC patients, we prospectively compared the relative percentages and other characteristics of circulating T cell subsets between SCLC patients and healthy controls.

The proportion of different subsets of circulating lymphocytes was alerted in cancer patients [23, 24]. Over the past decade, the increase of regulatory CD4^+^CD25^+^Foxp3^+^ Treg cells has been noticed in tumor-infiltrating lymphocytes from ovarian cancer and lung cancer [31]. Tregs is important to regulate immune response and preserve immune homeostasis. It mediates immunosuppression through the cell to cell contact or soluble inhibitory cytokines like transforming growth factor-β and IL-10 [32, 33]. It has been reported that the proportion of Tregs is elevated in circulating lymphocytes in patients with gastrointestinal malignant tumors, lung cancer and breast cancer [34]. In this study, we explored the percentages of different lymphocyte subsets to assess their potential role in predicting disease progression. Our study found the proportion of total CD3^+^ T cells and CD3^+^CD4^+^ T cells were decrease, paralleled with the increase of the percentage of Treg cells in SCLC patients. The increase of Tregs can result in immune tolerance and immunosuppression through down-regulating T cell signal molecules or inducing T cell apoptosis in cancer patients [31]. In addition, the elevation of Tregs may be related to tumor progression [31]. Although a higher proportion of Tregs was observed in SCLC patients than in healthy persons accomplish with a lower percentage of CD3^+^ total lymphocyte subsets, no prognostic value of these two subsets of lymphocyte was observed in this study.

One of the interesting findings from this study was that the proliferation of CD8^+^ T cells was concurrently lower in SCLC patients without significant changes in the percentage of CD8^+^ subsets. It is reported that CD8^+^ T cells play a key role in the clearance of intracellular pathogens and tumors [35]. However, high and persistent antigen and inflammatory stimulation can cause changes in CD8^+^ T cell differentiation or exhaustion in the process of chronic infection and tumor. T cell exhaustion is usually characterized by gradual and graded loss of effector function during persistent infection [36]. Normally, functions such as high proliferative capacity, IL-2 production, and cytokine polyfunctionality are lost early, then the production of chemokines like IFN-γ and the degranulation capacity is defective [17]. The loss of high proliferation ability is mainly found in tumor-infiltrating lymphocytes [17]. In our study, we also observed it in peripheral blood lymphocytes. According to the univariate analysis for PFS, the %divided CD8^+^ T cells positively correlated with PFS of SCLC patients. In the multivariate analyses, the higher %divided CD8^+^ T cells were proved to be an independent factor to predict a long PFS.

Although immune cells, especially T cells, play an effective role in immune surveillance and tumor growth control, inhibitory CD4^+^ and CD8^+^ regulatory T cells can be produced after chronic stimulation and interaction with tumor cells, which promote rather than inhibit the development and progress of the tumor [37]. The percentage of Tregs subsets was increased in our study, and we observed a negative correlation between it and the suppression of CD8^+^ proliferation. NLR and Platelet lymphocyte ratio (PLR) were reported associated with the alternation of peripheral lymphocytes and the prognosis of lung cancer patients [38]. However, we didn’t find a significant correlation between NLR and PLR with the proliferation of CD8^+^ T cells or the prognosis of patients in our study. The degree of chemotherapy or radiotherapy induced lymphopenia was correlated with the proliferation ability of CD8^+^ T cells in this study. It has been reported the exhaustion T cells are more sensitive to chemotherapy or radiotherapy [39]. The degree of lymphopenia may reflect the suppression of T cell function.

## Conclusions

In conclusion, our study indicates that patients with SCLC have impaired immunity in peripheral blood. The proliferation potential of CD8^+^ T cells in circulating lymphocytes is an independent predicator for PFS. Therefore, further study of the mechanism involved in the suppression of the proliferation of CD8^+^ lymphocytes in circulating lymphocytes may lead to the development of immunotherapy for SCLC patients.

## Supplementary information

**Additional file 1: Table S1.** Comparison for the healthy controls vs. patients’ cohort.

## Data Availability

All data generated or analyzed during this study are included in this published article.

## Abbreviations

ED-SCLC: extensive small cell lung cancer
Tregs: regulatory T cells
T_ex._: T cell exhaustion
PD-1: programmed death-1
PBMCs: peripheral blood mononuclear cells
CFSE: 5,6-carboxy fluorescein diacetate succinimide ester
NLR: neutrophil to lymphocyte ratio
PLR: platelet lymphocyte ratio

## References

[1] Herbst RS, Heymach JV, Lippman SM (2008). Lung cancer. N Engl J Med.

[2] Rudin CM, Ismaila N, Hann CL, Malhotra N, Movsas B, Norris K (2015). Treatment of small-cell lung cancer: American Society of Clinical Oncology Endorsement of the American College of Chest Physicians Guideline. J Clin Oncol.

[3] Travis WD (2012). Update on small cell carcinoma and its differentiation from squamous cell carcinoma and other non-small cell carcinomas. Modern Pathol..

[4] Simon GR, Turrisi A (2007). Management of small cell lung cancer: ACCP evidence-based clinical practice guidelines. Chest.

[5] Byers LA, Rudin CM (2015). Small cell lung cancer: where do we go from here?. Cancer.

[6] Puglisi M, Dolly S, Faria A, Myerson JS, Popat S, O’Brien ME (2010). Treatment options for small cell lung cancer—do we have more choice?. Br J Cancer.

[7] Spigel DR, Socinski MA (2013). Rationale for chemotherapy, immunotherapy, and checkpoint blockade in SCLC: beyond traditional treatment approaches. J Thor Oncol.

[8] Darnell RB, DeAngelis LM (1993). Regression of small-cell lung carcinoma in patients with paraneoplastic neuronal antibodies. Lancet (London, England)..

[9] Nakamura H, Saji H, Ogata A, Hosaka M, Hagiwara M, Kawasaki N (2002). Immunologic parameters as significant prognostic factors in lung cancer. Lung Cancer..

[10] Wang W, Hodkinson P, McLaren F, MacKinnon A, Wallace W, Howie S (2012). Small cell lung cancer tumour cells induce regulatory T lymphocytes, and patient survival correlates negatively with FOXP3+ cells in tumour infiltrate. Int J Cancer.

[11] Hanahan D, Weinberg RA (2011). Hallmarks of cancer: the next generation. Cell..

[12] Dunn GP, Old LJ, Schreiber RD (2004). The three Es of cancer immunoediting. Annu Rev Immunol.

[13] Topalian SL, Drake CG, Pardoll DM (2015). Immune checkpoint blockade: a common denominator approach to cancer therapy. Cancer Cell.

[14] Topalian SL, Taube JM, Anders RA, Pardoll DM (2016). Mechanism-driven biomarkers to guide immune checkpoint blockade in cancer therapy. Nat Rev Cancer.

[15] Gallimore A, Glithero A, Godkin A, Tissot AC, Plückthun A, Elliott T (1998). Induction and exhaustion of lymphocytic choriomeningitis virus–specific cytotoxic T lymphocytes visualized using soluble tetrameric major histocompatibility complex class I–peptide complexes. J Exp Med.

[16] Lee PP, Yee C, Savage PA, Fong L, Brockstedt D, Weber JS (1999). Characterization of circulating T cells specific for tumor-associated antigens in melanoma patients. Nat Med.

[17] Kurachi M (2019). CD8+ T cell exhaustion. Seminars in immunopathology.

[18] Pauken KE, Wherry EJ (2015). Overcoming T cell exhaustion in infection and cancer. Trends Immunol.

[19] Barber DL, Wherry EJ, Masopust D, Zhu B, Allison JP, Sharpe AH (2006). Restoring function in exhausted CD8 T cells during chronic viral infection. Nature.

[20] Gros A, Parkhurst MR, Tran E, Pasetto A, Robbins PF, Ilyas S (2016). Prospective identification of neoantigen-specific lymphocytes in the peripheral blood of melanoma patients. Nat Med.

[21] Tahara H, Ide K, Basnet N, Tanaka Y, Ohdan H (2010). Determination of the precursor frequency and the reaction intensity of xenoreactive human T lymphocytes. Xenotransplantation.

[22] Tanaka Y, Ohdan H, Onoe T, Asahara T (2004). Multiparameter flow cytometric approach for simultaneous evaluation of proliferation and cytokine-secreting activity in T cells responding to allo-stimulation. Immunol Invest.

[23] Lau KM, Cheng SH, Lo KW, Lee SAKW, Woo JKS, van Hasselt CA (2007). Increase in circulating Foxp3+CD4+CD25high regulatory T cells in nasopharyngeal carcinoma patients. Br J Cancer.

[24] Hu X, Gu Y, Zhao S, Hua S, Jiang Y (2019). Elevated circulating CD4(+)CD25(−)Foxp3(+) regulatory T cells in patients with nonsmall cell lung cancer. Cancer Biother Radiopharm..

[25] Tiwari M (2010). From tumor immunology to cancer immunotherapy: miles to go. J Cancer Res Ther..

[26] Kinoshita T, Muramatsu R, Fujita T, Nagumo H, Sakurai T, Noji S (2016). Prognostic value of tumor-infiltrating lymphocytes differs depending on histological type and smoking habit in completely resected non-small-cell lung cancer. Ann Oncol.

[27] Marshall EA, Ng KW, Kung SH, Conway EM, Martinez VD, Halvorsen EC (2016). Emerging roles of T helper 17 and regulatory T cells in lung cancer progression and metastasis. Mol Cancer..

[28] Fu J, Xu D, Liu Z, Shi M, Zhao P, Fu B (2007). Increased regulatory T cells correlate with CD8 T-cell impairment and poor survival in hepatocellular carcinoma patients. Gastroenterology.

[29] Holcombe RF, Jacobson J, Dakhil SR, Stewart RM, Betzing KS, Kannan K (1999). Association of immune parameters with clinical outcome in stage III colon cancer: results of Southwest Oncology Group Protocol 9009. Cancer Immunol Immunother.

[30] Vesely P, Touskova M, Melichar B (2005). Phenotype of peripheral blood leukocytes and survival of patients with metastatic colorectal cancer. Int J Biol Markers..

[31] Woo EY, Chu CS, Goletz TJ, Schlienger K, Yeh H, Coukos G (2001). Regulatory CD4(+)CD25(+) T cells in tumors from patients with early-stage non-small cell lung cancer and late-stage ovarian cancer. Cancer Res.

[32] Sakaguchi S (2005). Naturally arising Foxp3-expressing CD25+CD4+ regulatory T cells in immunological tolerance to self and non-self. Nat Immunol.

[33] Chen W, Jin W, Hardegen N, Lei K-J, Li L, Marinos N (2003). Conversion of peripheral CD4+CD25− naive T cells to CD4+CD25+ regulatory T cells by TGF-β induction of transcription factor Foxp3. J Exp Med..

[34] Okita R, Saeki T, Takashima S, Yamaguchi Y, Toge T (2005). CD4+CD25+ regulatory T cells in the peripheral blood of patients with breast cancer and non-small cell lung cancer. Oncol Rep.

[35] Kaech SM, Cui W (2012). Transcriptional control of effector and memory CD8+ T cell differentiation. Nat Rev Immunol.

[36] Wherry EJ, Kurachi M (2015). Molecular and cellular insights into T cell exhaustion. Nat Rev Immunol.

[37] Wang HY, Wang RF (2007). Regulatory T cells and cancer. Curr Opin Immunol.

[38] Chowdhary M, Switchenko JM, Press RH, Jhaveri J, Buchwald ZS, Blumenfeld PA (2018). Post-treatment neutrophil-to-lymphocyte ratio predicts for overall survival in brain metastases treated with stereotactic radiosurgery. J Neurooncol.

[39] Onyema OO, Decoster L, Njemini R, Forti LN, Bautmans I, De Waele M (2015). Chemotherapy-induced changes and immunosenescence of CD8+ T-cells in patients with breast cancer. Anticancer Res.

