# Supplementary material for: The prognostic role of circulating CD8+ T cell proliferation in patients with untreated extensive stage small cell lung cancer
Source: J Transl Med. 2019 Dec 3;17:402. doi: 10.1186/s12967-019-02160-7 (PMC6892179; doi:10.1186/s12967-019-02160-7)
Supplement: Supplementary file 1 — Additional file 1: Table S1. Comparison for the healthy controls vs. patients’ cohort. [file 12967_2019_2160_MOESM1_ESM.docx]

Additional file 1: Table S1. Comparison for the healthy controls vs. patients’ cohort

| Item | Patients cohort  (N = 36) | Healthy controls  (N = 22) | *P* value |
| --- | --- | --- | --- |
| CD3^+^ subsets % | 41.9 ± 14.5 | 55.6 ± 11.8 | <0.001 |
| CD3^+^CD4^+^ subsets % | 27.0 ± 9.43 | 33.5 ± 5.08 | 0.0014 |
| CD3^+^CD8^+^ subsets % | 30.9 ± 12.8 | 28.8 ± 9.97 | 0.4914 |
| CD4^+^ CD25^+^ Foxp3^+^ subsets % | 6.11[2.13-13.5] | 3.8[2.74-8.30] | 0.0083 |
| CD4^+^ %divided | 85.2[68.6-97.9] | 88.8[75.6-93.4] | 0.1611 |
| CD8^+^ %divided | 85.3[62.9-96.3] | 91.3[82.9-96.9] | 0.0058 |

*P* values were calculated by Student’s t test or the Mann-Whitney U test.

Data were presented as mean values ± standard deviation or medium[range].
